# Prenatal diagnosis to postnatal outcomes in multicystic dysplastic kidney: experience of a tertiary center in the Black Sea region

**DOI:** 10.1590/1806-9282.20251175

**Published:** 2025-12-15

**Authors:** Gökhan Ünver, Sercan Serin, Miğraci Tosun, Handan Çelik, Mesut Önal, Ümmet Abur, Engin Altundağ

**Affiliations:** 1 Ondokuz Mayis University, Faculty of Medicine, Department of Obstetrics and Gynecology, Perinatology Unit – Samsun, Turkey.; 2 Ondokuz Mayis University, Faculty of Medicine, Department of Obstetrics and Gynecology – Samsun, Turkey.; 3 Ondokuz Mayis University, Faculty of Medicine, Department of Medical Genetics – Samsun, Turkey.

**Keywords:** Antenatal care, Multicystic dysplastic kidney, Prenatal diagnosis

## Abstract

**OBJECTIVE:**

The aim of this study was to determine the association of unilateral multicystic dysplastic kidney in fetuses with genetic disorders, syndromic conditions, accompanying anatomical anomalies, and postnatal prognosis.

**METHODS:**

Cases diagnosed with multicystic dysplastic kidney and followed at the Perinatology Clinic of Samsun Ondokuz Mayıs University between January 2012 and June 2024 were retrospectively reviewed. Demographic, ultrasonographic, genetic, and postnatal outcomes were analyzed.

**RESULTS:**

Thirty-eight fetuses with multicystic dysplastic kidney were identified during intrauterine life. The diagnosis was made via antenatal ultrasonography in 97.2% of cases. Laterality was on the left side in 55.3% and on the right side in 44.7% of multicystic dysplastic kidney cases. The cohort consisted of 42.1% females and 57.9% males. Major extrarenal anomalies were present in 21.1% of fetuses with unilateral multicystic dysplastic kidney. Amniocentesis for karyotyping was performed in 23.7% of cases, all of which yielded normal karyotypes. Contralateral kidney anomalies were detected in 13.2% of cases, and ureterocele was observed in 15.8%. The most common postnatal anomalies were ureteropelvic junction obstruction and grade four-five vesicoureteral reflux. During a mean follow-up period of 7 years, no cases of hypertension or Wilms tumor were identified.

**CONCLUSION:**

Prenatal diagnosis of multicystic dysplastic kidney is crucial for early detection of potential contralateral kidney anomalies and predicting postnatal outcomes. In cases of isolated multicystic dysplastic kidney, the likelihood of karyotypic abnormalities, malignancies, or hypertension is very low, and the postnatal prognosis is favorable.

## INTRODUCTION

The diagnosis of multicystic dysplastic kidney (MCDK) in antenatal life is established by ultrasonographic identification of multiple hypoechoic, noncommunicating cysts replacing normal renal parenchyma. MCDK represents the most common cause of renal cystic disease in neonates, with a reported incidence of 1 in 4.300 live births^1^.

Multicystic dysplastic kidney is unilateral in approximately 75% of cases, with a predilection for the left kidney and male fetuses. Bilateral involvement is typically incompatible with life^2^.

Multicystic dysplastic kidney arises from congenital ureteral obstruction or malformation of the ureteric bud branches and ampulla during early nephrogenesis^3^.

Extrarenal malformations occur in approximately 25% of cases. Among these anomalies, cardiac, central nervous system, and gastrointestinal tract abnormalities are most frequently observed. The presence of extrarenal anomalies increases the likelihood of an underlying chromosomal or genetic disorder^4^.

The prevalence of genetic disorders and chromosomal abnormalities in fetuses with MCDK ranges from ­approximately 7 to 14%. The prognosis of MCDK depends primarily on contralateral kidney anomalies. The most common associated defect is contralateral vesicoureteral reflux (VUR), with a reported incidence ranging from 5 to 43%^5^.

The aim of our study is to investigate the association of cases diagnosed with unilateral MCDK in fetal life with syndromes, the coexistence with genetic disorders, potential anatomical anomalies, and the postpartum prognosis.

## METHODS

This retrospective study included 38 cases diagnosed with unilateral MCDK among patients referred to the Perinatology Clinic of Samsun Ondokuz Mayıs University between 2012 and 2024 for evaluation of suspected fetal anomalies. The study protocol received approval from the institutional ethics committee (Approval number: 2024-384).

The patients’ medical histories, ultrasonography reports, imaging findings, genetic test results, delivery outcomes, and postnatal follow-up data were obtained from the hospital database. For cases managed outside our instution, postnatal prognosis and follow-up data were collected through telephone interviews.

**Figure 1:**
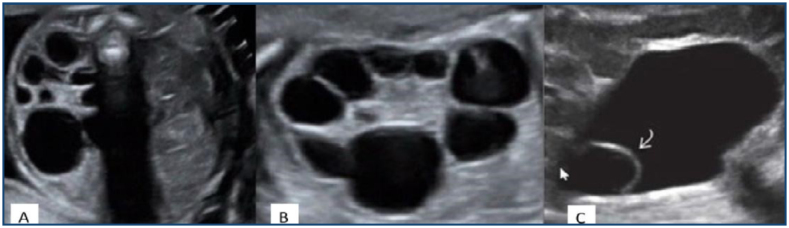
(A) Axial ultrasound image showing a left multicystic dysplastic kidney. (B) Coronal view demonstrating multiple noncommunicating cysts with no identifiable renal parenchyma, consistent with multicystic dysplastic kidney. (C) A cystic, anechoic, thin-walled ureterocele within the bladder.

The diagnosis of MCDK was based on ultrasonographic findings, including loss of corticomedullary differentiation, irregular contours, and the presence of multiple noncommunicating (unconnected) cysts of varying sizes (Figure 1).

Both fetal kidneys were identified in longitudinal and axial planes. Renal dimensions and the anteroposterior (AP) diameters of the renal pelvis were measured. Pelviectasis was defined as an AP diameter of the renal pelvis ≥4 mm before 28 weeks of gestation and ≥7 mm after 28 weeks. In cases diagnosed with MCDK, the presence of a ureterocele was described as a cystic, anechoic, thin-walled structure within the bladder.

The amniotic fluid volume was assessed by measuring the single deepest vertical pocket of <2 cm was defined as oligohydramnios.

Continuous variables were expressed as mean±standard deviation.

## RESULTS

In this retrospective study, 38 cases diagnosed with unilateral MCDK were included. At the time of diagnosis, the mean maternal age was 26.94±5.89 years, and the mean gestational age at diagnosis was 24.85±3.84 weeks. Evaluation was performed after 28 weeks of gestation in 23.6% of the cases. Amniotic fluid volume was within normal limits in 94.7% of cases, whereas oligohydramnios was identified in 5.3%. Laterality was on the left side in 55.3% and on the right side in 44.7% of MCDK cases.

Contralateral renal anomalies were identified in 13.2% of cases. Among these, four cases exhibited renal pyelectasis, while one case presented with a duplex collecting system. Ureterocele was detected in 15.8% of cases.

Major extrarenal anomalies were present in 21.1% of fetuses with unilateral MCDK, including: severe ventriculomegaly (one case), tracheoesophageal fistula (one case), ambiguous genitalia (one case), tetralogy of Fallot (one case), cleft lip/palate (one case), congenital pulmonary airway malformation type 3 (one case), hypospadias with single umbilical artery (one case), and hemivertebra with congenital scoliosis (one case).

Amniocentesis for karyotyping was performed in 23.7% of cases. Three of these cases had associated extrarenal anomalies: one with tetralogy of Fallot and single umbilical artery, one with tracheoesophageal fistula, and one with congenital pulmonary airway malformation type 3 in the right lung. The remaining six cases were classified as isolated unilateral MCDK. All karyotype analyses yielded normal results.

Postnatal follow-up was achieved in 36 of 38 cases (94.7%), with two pregnancies terminated due to associated tetralogy of Fallot and severe ventriculomegaly. Postnatal outcomes were documented for all 36 live-born cases.

During postnatal follow-up, one case initially diagnosed as MCDK was reclassified as medullary sponge kidney. The prenatal ultrasonographic diagnostic accuracy was 97% (35/36 correct diagnoses). Delivery modes included vaginal birth in 11 cases (28.9%) and cesarean section in 27 cases (71.1%).

The mean gestational age at delivery was 37 weeks and 6 days, with a mean birth weight of 3,201±538 grams. The clinical and demographic characteristics of the cases are summarized in Table 1.

**Table 1:** Antenatal demographic, sonographic, and clinical characteristics of the 38 cases.

Characteristics	Median (range) or n/N (%)
Maternal age at diagnosis	26.94±5.89
Gestational age at diagnosis	24.85±3.84 (17–32)
Foetal sex	16 female (42.1%) 22 male (57.9%)
Amnion fluid index	2 oligohydramnios (5.3%) 36 normal (94.7%)
Laterality (left/right side)	21 left (55.3%) 17 right (44.7%)
Ureterocele	6 (15.8%)
Major extrarenal anomaly	8 (21.1%) 1 Tetralogy of Fallot with single umblical artery 1 Ambiguous genitalia 1 Tracheoesophageal fistula 1 Severe ventriculomegaly 1 Congenital pulmonary airway malformation type 3 1 Hemivertebra with congenital scoliosis 1 Cleft lip/palate 1 Hypospadias with single umbilical artery
The anomaly of contralateral kidney	5 (13.2%)
Karyotype analysis	9 (23.7%)
Termination of pregnancy	2 (5.2%)
Gestational age at birth (weeks)	37 week 6 day (34 week 4 day-40 week 1 day)
Birthweight (gram)	3,201±538 g (1,638–4,170 g)
Route of delivery	Vaginal birth:11 (28.9%) Cesarean birth 27 (77.1%)

The mean follow-up period was 7±3.83 years. Accompanying congenital genitourinary anomalies are summarized in Table 2, with contralateral renal anomalies detected postnatally in five cases (13.9%). Grade four-five VUR was identified in two cases (5.6%), both of whom were male and underwent ureteroneocystostomy following stinging. UPJO was found in three cases (8.3%), all of whom were male, with two requiring pyeloplasty.

**Table 2:** Postnatal findings and outcomes of 36 cases.

Postnatal findings	Median (range) or n/N (%)
Follow-up interval (year)	7±3.83 (1–13)
Contralateral kidney anomaly	5 (13.9%)
Ureteronephrectomy	1 (2.8%)
Vesicourethral reflux (Grade four-five)	2 (5.6)
Ureteropelvic junction obstruction	3 (8.3%)
Ureterocele	6 (16.7%)
OHVIRA syndrome (Obstructed hemivagina with ipsilateral renal agenesis following spontaneous regression of MCDK, associated with a double uterus)	1 (2.8%)
Hypospadias	1 (2.8%)
Undescended testis	2 (5.6%)
Inguinal hernia	1 (2.8%)
Hearing loss	1 (2.8%)

MCDK: multicystic dysplastic kidney.

Ureterocele was detected in six cases (16.7%), with only one case requiring surgical intervention due to the development of stage four-five VUR. Bladder outlet obstruction was not observed prenatally in any of our six cases. All six cases were incidentally male, and none were associated with a duplex collecting system. One case presented with MCDK and ureterocele accompanied by tracheoesophageal fistula. The patient, now five years old, had undergone surgeries for tracheoesophageal fistula, experienced epileptic seizures, and exhibited additional problems such as developmental delay, speech impairment, and inability to walk without support.

A ureteroneohrectomy was performed in a single case (2.8%), representing the first patient diagnosed in our cohort in 2012. In one case, meatoplasty was performed due to hypospadias. Two cases were diagnosed with undescended testes and underwent surgical correction. One case exhibited a genital anomaly identified as OHVIRA syndrome (obstructed hemivagina with ipsilateral renal agenesis following spontaneous regression of MCDK, associated with a double uterus). Prenatal suspicion of ambiguous genitalia was noted in this case, but karyotyping revealed 46-XX. One patient received cochlear implantation due to hearing loss. One case underwent surgery for hemivertebra and congenital scoliosis, while another required postnatal surgery for tracheoesophageal fistula. The postnatal findings and outcomes of 36 cases are summarized in Table 2.

## DISCUSSION

Ultrasonography is the primary and most valuable imaging modality for diagnosing MCDK, both prenatally and postnatally. Diagnosis is typically established during routine obstetric ultrasonography in the antenatal period, enabling postnatal management planning^5^. Turkyilmaz et al. reported a prenatal detection rate of 97.3% in 144 cases of unilateral MCDK^6^. In our series, postnatal follow-up confirmed MCDK in 35 of 36 patients (97.2%), further validating the high reliability of prenatal ultrasonography for MCDK evaluation.

When unilateral MCDK is diagnosed prenatally, the assessment of the contralateral kidney and other fetal anatomical anomalies is crucial for counseling. A meta-analysis by Schreuder et al. found contralateral renal anomalies in approximately one-third of cases, with VUR accounting for one-fifth of these anomalies^7^. In our study, contralateral renal anomalies were detected in 14.0% of cases, including two with grade four-five VUR (requiring surgery) and three with UPJO. Given that children with MCDK rely on a solitary functioning kidney and face potential renal functional decline, early detection of contralateral anomalies is critical for determining pediatric nephrology follow-up frequency and prognostic outcomes.

The prevalence of extrarenal anomalies in MCDK is ∼25%, warranting detailed fetal organ screening^1^. In our cohort, extrarenal anomalies were identified in 21.1% of cases, none of which were interrelated. Karyotypic abnormalities are more likely in MCDK with extrarenal defects or bilateral involvement. Turkyilmaz et al. observed trisomy 18 in only two of 144 fetuses with extrarenal anomalies, while no karyotypic abnormalities were found in isolated MCDK cases^6^. Similarly, Lazebnik et al. reported no chromosomal anomalies in isolated unilateral MCDK^8^. Our study also revealed no karyotypic abnormalities. However, the chromosomal microarray analysis has identified pathogenic copy number variants in 14% of such cases^9^. For nonisolated MCDK, syndrome-specific molecular testing may be indicated.

The literature suggests excellent prognosis for prenatally diagnosed ureteroceles associated with MCDK^10^. We detected ureterocele in six cases (16.7%), with only one requiring surgery for stage four-five VUR. Ureterocele rarely coexists with other systemic anomalies but may cause intrauterine obstruction leading to oligohydramnios^10^.

Kopač et al. reported no hypertension in 80 children with MCDK^11^. The primary risk factor for hypertension is contralateral renal injury, emphasizing the need for ambulatory blood pressure monitoring in children with contralateral anomalies^12^. None of our patients developed postnatal hypertension, likely due to early prenatal diagnosis and rigorous contralateral kidney surveillance.

The largest study on Wilms tumor risk in MCDK patients estimated an incidence of 1:2,000^13^. No cases were observed in our cohort during a mean 7-year follow-up, supporting minimal malignant transformation risk. Consequently, nephrectomy indications for MCDK have become increasingly restricted, with a significant decline in incidence.

In the study conducted by Kiyak et al., genital anomalies were observed in 3–13% of children diagnosed with MCDK, whereas our series revealed genital anomalies in 14.0% of cases^14^. We identified undescended testes in two cases, OHVIRA syndrome in one case, hypospadias in one case, and inguinal hernia in one case. Therefore, careful and detailed postnatal urogenital screening should be recommended for patients with MCDK.

One patient with MCDK and hearing loss underwent cochlear implantation. Branchio-oto-renal (BOR) syndrome, an autosomal dominant disorder affecting the embryonic development of branchial arches, otic structures, and the renal system, was prenatally diagnosed by Tang et al. via cordocentesis revealing an EYA1 mutation^15^. Prenatal diagnosis facilitates parental counseling and informed decision-making. Further research is needed to elucidate intrauterine manifestations and genetic underpinnings of BOR syndrome.

## LIMITATIONS

This study has several limitations that should be considered when interpreting its findings. The most significant limitation is the relatively small sample size, which is inherent to its design as a single-center, longitudinal cohort study. This restricts the statistical power of our analyses and the generalizability of our results to broader populations. Furthermore, the study's retrospective nature introduces the potential for selection bias and unmeasured confounding variables. While our follow-up period was long for included cases, the small cohort size limited our ability to perform robust subgroup analyses for each specific anomaly or genetic subtype. Future multicenter, prospective studies with larger patient cohorts are necessary to validate our findings and provide more powerful statistical conclusions.

## CONCLUSION

Our study demonstrates that prenatal ultrasonography exhibits high sensitivity in diagnosing MCDK. The absence of hypertension or Wilms tumor in our cohort suggests that routine nephrectomy and aggressive screening protocols may be unnecessary in isolated MCDK, supporting a conservative management approach.

The higher need for surgical intervention in male fetuses underscores the importance of detailed prenatal urinary tract evaluation in this population. Postnatal follow-up revealed compensatory hypertrophy and preserved function of the contralateral kidney, indicating sufficient renal reserve. These findings imply that monitoring the contralateral kidney may suffice in isolated MCDK, avoiding unnecessary invasive procedures.

No karyotypic abnormalities were detected in isolated MCDK cases, challenging the necessity of routine invasive testing. However, the potential association with rare syndromes (e.g., BOR syndrome) warrants targeted genetic evaluation when additional anomalies are present.

## Data Availability

The datasets generated and/or analyzed during the current study are available from the corresponding author upon reasonable request.

## References

[1] Chetty S, Society for Maternal-Fetal Medicine (SMFM) (2021). Multicystic dysplastic kidney. Am J Obstet Gynecol.

[2] Raina R, DeCoy M, Chakraborty R, Mahajan S, Moran R, Gibson K (2021). Renal cystic diseases during the perinatal and neonatal period. J Neonatal Perinatal Med.

[3] Raina R, Chakraborty R, Sethi SK, Kumar D, Gibson K, Bergmann C (2021). Diagnosis and management of renal cystic disease of the newborn: core curriculum 2021. Am J Kidney Dis.

[4] Çaltek HÖ, Çaltek NÇ, Aras D, Çolak TNÇ, Okşen E, Yavuz S (2025). Prenatal diagnosis and postnatal outcomes of congenital kidney and urinary tract anomalies: results from a tertiary center. BMC Pregnancy Childbirth.

[5] Rosenblum ND, Gupta IR (2023). Pediatric kidney disease.

[6] Turkyilmaz G, Cetin B, Erturk E, Sivrikoz T, Kalelioglu I, Has R (2021). Prenatal diagnosis and outcome of unilateral multicystic kidney. J Obstet Gynaecol.

[7] Schreuder MF, Westland R, Wijk JA (2009). Unilateral multicystic dysplastic kidney: a meta-analysis of observational studies on the incidence, associated urinary tract malformations and the contralateral kidney. Nephrol Dial Transplant.

[8] Lazebnik N, Bellinger MF, Ferguson JE, Hogge JS, Hogge WA (1999). Insights into the pathogenesis and natural history of fetuses with multicystic dysplastic kidney disease. Prenat Diagn.

[9] Xi Q, Zhu X, Wang Y, Ru T, Dai C, Wang Z (2016). Copy number variations in multicystic dysplastic kidney: update for prenatal diagnosis and genetic counseling. Prenat Diagn.

[10] Turkyilmaz G, Cetin B, Sivrikoz T, Erturk E, Oktar T, Kalelioglu I (2019). Antenatally detected ureterocele: associated anomalies and postnatal prognosis. Taiwan J Obstet Gynecol.

[11] Kopač M, Kordič R (2022). Associated anomalies and complications of multicystic dysplastic kidney. Pediatr Rep.

[12] Seeman T, John U, Bláhová K, Vondrichová H, Janda J, Misselwitz J (2001). Ambulatory blood pressure monitoring in children with unilateral multicystic dysplastic kidney. Eur J Pediatr.

[13] Homsy YL, Anderson JH, Oudjhane K, Russo P (1997). Wilms tumor and multicystic dysplastic kidney disease. J Urol.

[14] Kiyak A, Yilmaz A, Turhan P, Sander S, Aydin G, Aydogan G (2009). Unilateral multicystic dysplastic kidney: single-center experience. Pediatr Nephrol.

[15] Tang P, Li J, Li J, Yang J, Zhu J (2022). Prenatal diagnosis and genetic analysis of a fetus with Branchio-oto-renal syndrome: a case report. Medicine.

